# From planning to practice: building the national network for the surveillance of severe maternal morbidity

**DOI:** 10.1186/1471-2458-11-283

**Published:** 2011-05-08

**Authors:** Samira M Haddad, José G Cecatti, Mary A Parpinelli, João P Souza, Maria L Costa, Maria H Sousa, Fernanda G Surita, João L Pinto e Silva, Rodolfo C Pacagnella, Rodrigo S Camargo, Maria V Bahamondes, Vilma Zotareli, Lúcio T Gurgel, Lale Say, Robert C Pattinson

**Affiliations:** 1 Department of Obstetrics and Gynecology, School of Medical Sciences, University of Campinas, Campinas, Brazil; 2 Campinas Center for Studies in Reproductive Health (CEMICAMP), Campinas, Brazil; 3 UNDP/UNFPA/WHO/World Bank Special Programme of Research, Development and Research Training in Human Reproduction, Department of Reproductive Health and Research, World Health Organization, Geneva, Switzerland; 4 MRC Maternal and Infant Health Care Strategies Research Unit, University of Pretoria, Gauteng, South Africa

**Keywords:** surveillance network, severe maternal morbidity, near-miss, multicenter cross-sectional study

## Abstract

**Background:**

Improving maternal health is one of the Millennium Development Goals for 2015. Recently some progress has been achieved in reducing mortality. On the other hand, in developed regions, maternal death is a relatively rare event compared to the number of cases of morbidity; hence studying maternal morbidity has become more relevant. Electronic surveillance systems may improve research by facilitating complete data reporting and reducing the time required for data collection and analysis. Therefore the purpose of this study was to describe the methods used in elaborating and implementing the National Network for the Surveillance of Severe Maternal Morbidity in Brazil.

**Methods:**

The project consisted of a multicenter, cross-sectional study for the surveillance of severe maternal morbidity including near-miss, in Brazil.

**Results:**

Following the development of a conceptual framework, centers were selected for inclusion in the network, consensus meetings were held among the centers, an electronic data collection system was identified, specific software and hardware tools were developed, research material was prepared, and the implementation process was initiated and analyzed.

**Conclusion:**

The conceptual framework developed for this network was based on the experience acquired in various studies carried out in the area over recent years and encompasses maternal and perinatal health. It is innovative especially in the context of a developing country. The implementation of the project represents the first step towards this planned management. The system online elaborated for this surveillance network may be used in further studies in reproductive and perinatal health.

## Background

The reduction of maternal mortality is one of the targets of the Millennium Development Goals for 2015 [1]. In some countries, some progress has been achieved, but there is very little progress in the most of high mortality countries [2-4].

The high mortality ratios result mainly from difficulties in accessing healthcare services, the inadequate management of obstetrical complications and failure to provide effective interventions in poorly developed areas [5]. On the other hand, the occurrence of maternal death in developed settings is a relatively rare event compared to the total number of women who survive such complications [3]. The study of severe maternal morbidity has been suggested as a useful approach to investigating quality of health care systems in order to improve women's healthcare and effectively reduce maternal morbidity [5]. Nevertheless, differences also exist in the definitions and procedures used to identify cases of morbidity, which need also progressive transformation and development [6,7]. Hospital-based and population-based studies have shown that lack of standardization of the criteria used to define severe maternal morbidity, difficulty in identifying and reporting these conditions both with official records and by the women themselves, and the limitations of retrospectively conducted studies [8-14].

Electronic surveillance systems may introduce improvements in the process by facilitating complete data reporting and reducing the time required for data collection and analysis [15-18]. With the objective of providing support for healthcare programs, epidemiological surveillance systems may be defined as "the ongoing and systematic collection, analysis and interpretation of health data in the process of describing and monitoring a health event" [19]. In improving healthcare, greater benefits are obtained when an integrated system of data technology is available and if this systematic electronic data capture system is associated with a program to identify risks and propose clinical management based on evidence [20]. Few countries and institutions have well-structured systems of health data technology in which data are used in real-time for adjusting healthcare and performing surveillance [21-25].

Even in places where the health surveillance system is adequately structured such as in Canada, severe maternal morbidity is not yet fully studied due to various factors: the need to standardize the concepts, the range of the area in which surveillance has to be carried out and the prospective individual evaluation of each identified case, with effective feedback conveyed to the healthcare providers [25].

In Brazil, the distribution of maternal death is associated with disparities in socioeconomic development. Brazil's large territorial extension is also associated with cultural differences and socioeconomic inequalities, resulting in heterogeneity with respect to the incidence of complications and in the ways of dealing with them [26]. Health-related data systems are almost exclusively used for epidemiological evaluation and global management, and are not integrated into a specific prospective evaluation of care.

Only a few initiatives for the surveillance of maternal mortality and severe maternal morbidity have been carried out prospectively [23,24]. As recently defined by the WHO, maternal near-miss refers to a situation in which a woman almost dies but survives a life-threatening complication of pregnancy, childbirth or in the first 42 days following delivery [5]. In order to facilitate the practical use of this concept, potentially life-threatening conditions were listed that, together with specific criteria defining maternal near-miss, would operationally characterize the broader concept of severe maternal morbidity.

As a result, the surveillance and proposal of strategies to reduce maternal deaths worldwide may be founded on a single conceptual basis. Therefore, the objective of the present manuscript was to describe the methods and procedures adopted for the creation and implementation of the National Network for Surveillance of Severe Maternal Morbidity in Brazil [27], covering all the regions of the country and using the new standardized criteria for maternal near miss recently defined by the WHO [5].

## Methods

### Protocol design

In 2002, research was initiated at the University of Campinas, Brazil, focusing on severe maternal morbidity. The transition from studying death to studying maternal morbidity followed a worldwide trend, considering the absolute number of deaths is relatively small compared to the number of cases of morbidity. Data on maternal morbidity are more accessible and reliable for the evaluation of quality in obstetrical care. Within this scope, a study was conducted to evaluate the applicability of different concepts of severe maternal morbidity and of a severity score to identify cases of maternal morbidity [9].

Elaborating further on the concept that routine health data would be useful for systematically identifying the occurrence of complications associated with pregnancy, the National Health Service's Hospital Information System was evaluated. Data routinely collected from medical records of women with conditions suggestive of severe maternal morbidity were selected, and the diagnoses and procedures used in such cases were described in order to identify factors associated with the occurrence of maternal death [15]. Next, further evaluations on maternal morbidity were performed using data from demographic health surveys. The importance of the use of validated questionnaires for obtaining information on morbidity and the regional differences in the prevalence of morbidity were also highlighted [11].

Considering that the early identification of cases of maternal morbidity would allow a more appropriate way of monitoring, managing and preventing deaths [28], the proposal to establish the National Network for Surveillance of Severe Maternal Morbidity was developed as a research proposal [27].

### Organization of the project

The project is a multicenter, cross-sectional study to be implemented in referral obstetrical units in all geographical regions of Brazil. Over a 12-month period, prospective surveillance and data collection was planned to be performed to identify cases of maternal near-miss and potentially life-threatening conditions in accordance with the new criteria defined by WHO [5].

To determine the number of collaborating centers to be included in the study, sample size was calculated according to the number of deliveries that would have to be covered to identify cases of near-miss. Based on a previously reported incidence of 8 cases for 1000 deliveries [9], approximately 70,000 deliveries would have to be monitored. This number was believed to be sufficient to validate the new criteria issued by WHO [5]. The study population is composed of all the women admitted to the participating hospitals during the study period who suffer organ dysfunction (that will be a near-miss case or a maternal death, Table 1) or presenting potentially life-threatening conditions (Table 2), who die or are transferred to other healthcare services because they require more specialized services or procedures.

**Table 1:** Potentially life-threatening maternal conditions

HEMORRHAGIC COMPLICATIONS	
Abruptio placentae Placenta previa/accreta/increta/percreta Ectopic pregnancy Ruptured uterus Severe hemorrhage due to abortion	Postpartum hemorrhage Atony Retained placenta Perineal lacerations Coagulopathy Uterine inversion
**HYPERTENSIVE DISORDERS**	
Severe preeclampsia Eclampsia Hypertensive encephalopathy	Severe hypertension HELLP syndrome Acute fatty liver of pregnancy
**OTHER COMPLICATIONS**	
Pulmonary edema Seizures Sepsis Postpartum endometritis Post abortion endometritis Urinary infection Chest infection Thrombocytopenia < 100 000 platelets Thyroid crisis Shock	Acute respiratory failure Acidosis Cardiopathy Cerebrovascular accident Coagulation disorders Thromboembolism Diabetic ketoacidosis Jaundice/hepatic dysfunction Meningitis Acute renal failure
**MANAGEMENT INDICATORS OF SEVERITY**	
Transfusion of blood derivatives Central venous access ICU admission Prolonged hospital stay (> 7 days)	Intubation unrelated to anaesthesia Return to operating theater Major surgical intervention (hysterectomy, laparotomy) Use of magnesium sulfate

**Table 2:** WHO criteria for maternal near miss^5^

**CLINICAL CRITERIA**	
Acute cyanosis *Gasping* Breathing rate > 40 or < 6 per minute Shock Oliguria unresponsive to fluids or diuretics Coagulation disorders/clotting failure	Loss of consciousness for ≥ 12 h Unconscious, no pulse/heartbeat Cerebrovascular accident Uncontrolled convulsions/total paralysis Jaundice concomitantly with preeclampsia
**LABORATORY CRITERIA**	
Oxygen saturation < 90% for > 60 minutes PaO2/FiO2 < 200 mmHg Creatinine ≥ 300 mmol/l or ≥ 3,5 mg/dL Bilirubin > 100 mmol/l or ≥ 6,0 mg/dL	pH < 7,1 Lactate > 5 Acute thrombocytopenia (< 50 000 platelets) Unconscious, presence of glucose and ketoacidosis in urine
**MANAGEMENT CRITERIA**	
Use of continuous vasoactive drug Postpartum or post abortion hysterectomy due to infection or hemorrhage Blood transfusion ≥ 5 units of red cell	Intubation and ventilation for a period ≥ 60 minutes, unrelated to anesthesia Dialysis for treatment of acute renal failure Cardiopulmonary resuscitation (CPR)

During the data collection period, at each participating hospital, local coordinators perform a daily review of all admitted women, looking for cases with any of the conditions indicative of severity (Table 2). The lists of patients with these diagnoses are sent for review and data collection following the patient's discharge from hospital, death or transfer to another hospital. Data unavailable from the record is obtained from the attending team. Data are collected on demographic and obstetrical characteristics, primary determinant of severe morbidity (the first complication in the chain of events that led to severe maternal morbidity), length of hospitalization, occurrence of any criteria of maternal near-miss, perinatal outcome and condition of the woman at discharge from hospital. The data are collected on a pre-coded form and are then sent electronically to the database. The manually completed forms are filed in such a way as to be easily accessible for inspection during technical quality control visits.

### Selection of the centers to constitute the network

After the general proposal was ready, a meeting was held during a national congress of the Brazilian Federation of Societies of Gynecology and Obstetrics in November 2007 where representatives of several healthcare institutions from around the country were present. The proposal to establish a National Network for Surveillance of Severe Maternal Morbidity was presented and those interested in participating applied for that.

Before the project could be implemented, the proposal was submitted for public funding and, following approval, an invitation letter was sent to all interested institutions, together with a summary of the planned objectives and methods. In addition, a form designed to obtain information on the characteristics of the collaborating center was also sent to the local investigator. Basically, it had information on identification and location of the institution, nature and complexity level of the hospital, population covered, number of beds in the maternity department, availability of resources for more specialized care (blood bank, obstetrical and neonatal intensive care units, specialist care for high risk pregnancies, availability of other medical or surgical specialities, ultrasonography, laboratory, anesthetists available round the clock, resources for the parenteral administration of antibiotics, oxytocin and magnesium sulphate, resources for general anesthesia, mechanical ventilation, cardiorespiratory resuscitation of adults and newborn infants, hysterectomy), number of deliveries performed annually (minimum number required above 1,000 deliveries/year), availability of broadband internet connection, data on the prevalence of some obstetrical interventions based on scientific evidence performed during delivery, and availability of written protocols of procedures in the service.

Additionally telephone contacts occurred between the principal investigator and the person responsible for the institution. As a result of these different approaches, 35 institutions from all over Brazil applied for participating in the study. Evaluation of their characteristics and geographical distribution led to the selection of 27 institutions that fulfilled all the inclusion criteria.

### Review of the criteria for severe maternal morbidity and data collection forms

Following selection of the centers, a meeting was held in August 2008 with the principal investigators from each center at the project headquarters in Campinas. At this time, a term of agreement was signed by all attendants to compose a Brazilian Network for Studies in Reproductive and Perinatal Health. The objective of this alliance was to proceed to develop further studies in the future in the matter, using the same multicenter strategy of achieving regional diversity in a developing country with continental extensions. The meeting lasted for two days when the research proposal was reviewed and discussed, the concepts of near miss and severe maternal morbidity were presented, the data collection forms were structured and the concept of developing an electronic data collection system was introduced. A copy of the proposal was provided to each center, to be evaluated and approved locally. The coordinating center had the research protocol approved by the local institutional review board (Committee of Ethics in Research from the School of Medical Sciences, University of Campinas - Approval letter CEP 097/2009), and then by the national IRB.

### Selection of the electronic research system

**Figure 1:**
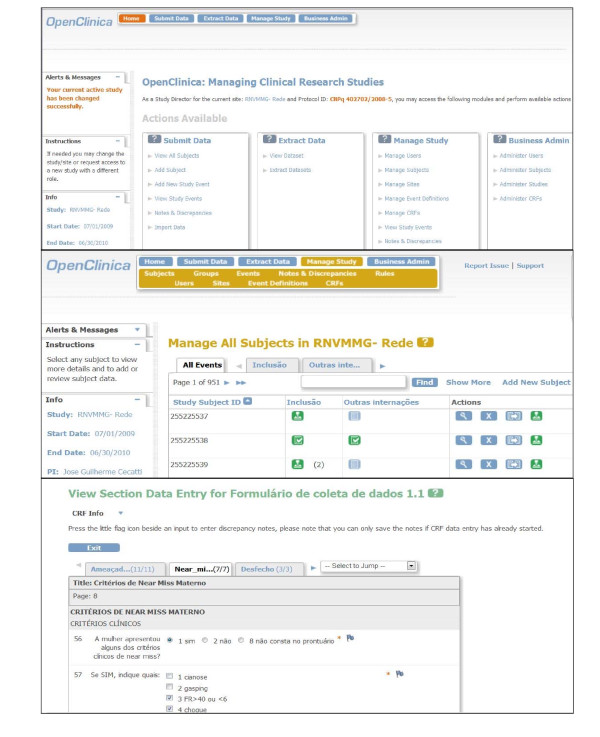
**Sample screens from the online data entry and management system:** A. Cover sheet for the study in OpenClinica; B. Form for the management of all subjects; C. Form for data entry on criteria for maternal near-miss.

The viability of the entire project depended on approval of the request for funding submitted to the National Research Council (CNPq)/Department of Science and Technology (DECIT). Initially, the plan was to develop software and a customized data management system control system for the study. Nevertheless, due to some practical constraints, it was decided to use a system that had already been developed and that would be cheaper to maintain. Therefore, a free, open source, online data entry system was selected (OpenClinica^®^) [29], which is available for use in clinical trials, was selected. This internet-based system consists of an electronic platform for data entry and management of data and is designed to support all types of clinical studies in a variety of locations [29]. The system permits autonomy in creating forms, in analyzing and storing data and in stratifying the right of access to be granted to users working in the same study (Figure 1).

## Results

### Development of specific software and hardware tools

Following selection of an electronic data entry system for the network and registration of the study in the OpenClinica^®^, an internet server was created in the host institution to safely store the data. The electronic address of the server was hosted in the institution's homepage with an individual safety certificate https://openclinica.caism.unicamp.br:8443/OpenClinica/MainMenu that allowed encrypted data to be sent to the central database (Figure 1). A detailed training was then carried out for the development of an electronic environment to serve the network. For this purpose, usernames and passwords were created for all research team, allowing individual access to their respective centers. Investigators, coordinators, supervisors, data managers at central and local levels were granted different levels of accessibility and privileges for the inclusion and evaluation of data. The electronic data collection form was developed in accordance with the standardized pattern offered by the system, with the inclusion of different sections containing all the variables pertinent to the study. Several versions had to be created and evaluated internally before the final version was reached.

### Development of material

In order to identify potential research subjects during hospitalization, an identification form was developed listing all the potentially life-threatening conditions. This form was produced and provided to the centers as a suggestion for use in selecting subjects at the moment of their discharge from hospital, mainly for hospitals with a large number of admissions. The manual data collection form was developed with exactly the same structure as the electronic version.

The manual of operations was designed to contain all the information required by the investigators and to provide well-structured material that could be easily and rapidly accessed. It contains the main concepts of the study, information on the participating centers and investigators, a detailed description of the basic steps involved in electronic data input and management and standardized definitions of the variables used in the study.

### Implementation process

With electronic data system and preliminary materials ready, a meeting was held in Campinas in April 2009 to present the system to all research teams and train them to use the the network. To assure the minimal material infrastructure required for the study, a computer was supplied to each center and training was provided using these same computers in an appropriate environment with internet support. The study documents and procedures were presented at the meeting and distributed to all the participants, who then discussed them and the electronic data system, making suggestions for changes in accordance with their individual experience at each site. The material was tested and personnel trained in its use through the presentation of clinical cases of maternal morbidity and mortality in order to recreate situations as close as possible to the actual routine expected for the study.

The meeting lasted three days and, in addition to practical training in the system, operational procedures were discussed to ensure that the study would function homogenously in the different sites of the network. It provided the opportunity to deal with a variety of aspects including defining concepts, routine procedures and the way the centers would operate. These debates resulted in changes that improved the instruments and standardized network operations.

Data collection was planned to start simultaneously in all centers, which happened at the beginning of July, 2009. The forms and manual of operations were provided to the investigators on a password-protected virtual disk, which is also hosted at the website, and contains the latest versions of all the documents used in the network.

### Analysis of the implementation process

After data collection was initiated, the process of data consistency checks and technical visits to the participating centers also started. Communication between all centers and the coordinating center was generally conducted by e-mail and telephone contact was seldom required. To verify the consistency of cases included in the system, a schedule was developed to be carried out at each individual center. During this procedure, all included cases are checked for inconsistencies by a team of trained research assistants in the coordinating center following a pretested protocol of general and specific consistencies between variables. Any errors or queries identified for any specific case are transmitted electronically to the local investigator and coordinator on a structured table. After evaluation and resolution of any inconsistencies, the local investigators return this table to the principal investigators, who conclude the audit by retaining, modifying or excluding the case.

Another quality control procedure that has been developed consists of technical visits to the centers, when evaluation is made of the working conditions of the equipment supplied, the appropriateness of the filing system used to store the manual forms, the use of the manual of operations and the particular strategies used to locally identify cases. A random check of selected patient forms is also made and the consistency of the data previously collected by the local investigators is verified. A report is then prepared for the local and central team. If a problem/situation arising from this visit is considered to be of general interest for all centers, a note is prepared and circulated among all research staff.

As initially planned, the availability of the professionals involved was crucial in controlling the network. In addition to the local staff, the existence in the coordinating center of a principal investigator, general and deputy coordinators, research assistants, network manager, system analyst, statistician, accounts manager and other technical assistants has proved to be essential for the follow-up of surveillance on such a broad scale.

## Discussion

The development of a prospective surveillance system for severe maternal morbidity in Brazil resulted in the National Network for the Surveillance of Severe Maternal Morbidity [27]. This is an innovative scientific initiative based on the experience acquired in the area by a research group on maternal morbidity and mortality at the coordinating center. In addition, this corresponds to the first time the new WHO criteria for maternal near miss will be prospectively used and validated. The full process was guaranteed by financial resources obtained from Brazilian funding agencies. These funds enabled the necessary infrastructure, including computers, the internet server, software, human resources to perform the surveillance and notification of data, the entire core organization of the study and the expenses involved in traveling to training meetings and technical visits. Nevertheless, these resources could be considered small taking into account the scale of the network structure, the complexity involved in controlling the quality of data collection and the duration of surveillance.

The decision to use an open data collection system specifically developed to support clinical studies rendered the implementation process less expensive and more practical. Although similar systems have already been used in developed countries to collect data on other subjects [16], to the best of our knowledge this is the first system developed for the prospective, widespread collection of data on severe maternal morbidity, thus permitting current epidemiological surveillance. More widespread analyses on the occurrence of severe maternal morbidity in Canada, for instance, were obtained using databases containing information routinely collected in healthcare services [25].

Meetings were of crucial importance for the development of a homogenous study. Situations differ greatly from one center to another as a result of their diverse geographical locations and resources available, although all of them were tertiary health facilities with neonatal intensive care units. Regarding their institutional capacity of providing appropriate care to obstetric complications, some of them are also provided with obstetrical ICU, some with general ICU and few have no ICU at all. The training allowed to update electronic and support material, a fruitful debate and the investigators to share their individual experiences. Communication between the centers, including discussions on problems and suggestions, was conducted by e-mail, ensuring a quick and cheap solution.

Data collection was initiated before the system could be tested by the investigators themselves in their own work environment. This resulted in the need to modify the form and the manual of operations after the first month of data collection. This may be considered a limitation in the planning and implementation of this study, highlighting the importance of pilot studies once the system is already fully operational in order to solve any difficulties or inconsistencies detected early. Despite that, all the updates required could be considered minor, involving completion of the electronic form and the definition of a few variables. Following these adjustments, no other changes have been required.

The manual of operations incorporated around 90% of the queries raised by the investigators prior to review and this efficacy increased following the modifications. The entire data entry procedure is described in detail there, including illustrations taken from the system itself for guidance. Nevertheless, many of the investigators sought advice before consulting the manual. This shows that reading instructions prior to initiating surveillance is a mandatory step to ensure that the process flows as effectively as possible.

Another possible limitation of the study would be that this kind of surveillance would identify only cases delivering in hospitals or health care facilities. However, nowadays, fortunately this is no longer a limitation in Brazil, considering the vast majority of deliveries occur in hospitals. Anyway, the new WHO criteria for identifying maternal near miss cases has a set of criteria that could theoretically be applied to any setting, even for community deliveries.

Currently the network has already finished its data collection's activities, with more than nine thousand and five hundred of cases of potentially life threatening and maternal near miss condition s included in the database, a number much higher than what was initially expected. The initiation of data collection coincided with the H1N1 influenza epidemic [30], which may have led to an increase in the occurrence of severe cases. Indeed, one of the changes made to the system was to add this diagnosis to the form.

Taking into account this partial experience, a next special concern arises on how to guarantee sustainability for a routine surveillance in a national environment. Considering that the process has showed to be more efficient in places where the form for identifying any potentially life threatening conditions or maternal near miss was routinely implemented, this should probably be an important content of a package directed to a nationwide system for surveillance of severe maternal morbidity. The development of a national electronic database system could facilitate the interpretation and management in different settings, by different professionals, and allow adaptation to local reality. To be more effective and complete, probably the surveillance might be a governmental strategy with scientific support by researchers and/or universities with expertise in the field. The Ministry of Health could enhance hospitals participation through supporting such surveillance as a public health policy. It could be first piloted as an official process in some facilities that had already participated in the current initiative, before a broader national implementation. The government could also enable adaptation of health information systems in use nowadays to the maternal morbidity surveillance needs.

Finally, there is an interesting point that appeared when this network first went into operation that should be the subject of a more in-depth qualitative investigation in a near future. Although this current project consists of a cross-sectional, observational study for the surveillance and detection of the occurrence of episodes of severe maternal morbidity in the participating centers, there have been emphatic reports from the network participants at each center that implementation and participation in this system has generated interventions that were not routine at these centers, including the use of some evidence-based interventions that had not yet been adopted (such as the routine prophylactic use of uterotonics at all deliveries), the review of the criteria of severity in obstetrical cases for referral to intensive care units and earlier request for specialist services to help manage cases in which specific dysfunctions and organ failure are detected, among others.

## Conclusions

The expectation generated following implementation of the National Network for Surveillance of Severe Maternal Morbidity is that it will lead to an increase in the production of knowledge on information technology and the surveillance of health events. The pioneering use of the criteria for near miss recently defined by the WHO [5] may permit validation of these criteria for later studies on a worldwide level. Hopefully other developing countries, and even developed countries, could implement similar surveillance systems and increase the consistency of data on maternal health. This increases the possibility of implementing actions that would indeed lead to a reduction in the unnecessary deaths of pregnant or postpartum women worldwide, as well as possibly also decreasing the burden of disease resulting from this condition for the many women who survive severe maternal morbidity. Therefore this initiative could complement the global strategy to reduce maternal mortality.

## Abbreviations

MDG: millennium development goal; WHO: World Health Organization

## Competing interests

The authors declare that they have no competing interests.

## Authors' contributions

The idea for the study arose in a group discussion among all the authors. The first version of the manuscript was drafted by SMH and JGC, and then complemented with the suggestions of the others. JGC supervised the entire process. All authors contributed to the development of the study protocol and approved the final version of the manuscript.

## Pre-publication history

The pre-publication history for this paper can be accessed here:

http://www.biomedcentral.com/1471-2458/11/283/prepub
